# In-Hospital Mortality in Patients with and without Dementia across Age Groups, Clinical Departments, and Primary Admission Diagnoses

**DOI:** 10.3390/brainsci14050455

**Published:** 2024-04-30

**Authors:** Karel Kostev, Bernhard Michalowsky, Jens Bohlken

**Affiliations:** 1 Epidemiology, IQVIA, 60549 Frankfurt, Germany; 2 University Clinic, Philipps-University, 35043 Marburg, Germany; 3 German Center for Neurodegenerative Diseases (DZNE), Site Rostock/Greifswald, Patient-Reported Outcomes & Health Economics Research, 17487 Greifswald, Germany; 4 Institute of Social Medicine, Occupational Health and Public Health, Faculty of Medicine, 04103 Leipzig, Germany

**Keywords:** dementia, Alzheimer’s disease, mortality, hospital, elderly

## Abstract

Background: Studies have reported higher in-hospital mortality rates in patients living with dementia (PlwD) with limited evidence across age groups, clinical departments, and admission diagnoses. The aim of this study was to compare the in-hospital mortality rate of PlwD with patients without dementia across groups, clinical departments, and admission diagnoses. Methods: This case-control study included patients aged ≥ 60 years hospitalized in 1 of 14 German hospitals between January 2019 and July 2023. PlwD were matched to patients without dementia. The associations between dementia and in-hospital mortality across groups were assessed using univariable logistic regression analyses. Results: 15,956 patients with and 15,956 without dementia were included (mean age: 83.9 years, 60.7% female). PlwD had a significantly higher in-hospital mortality rate (14.0% vs. 11.7%; OR 1.24, 95% CI: 1.16–1.32) than non-dementia controls. The highest excess mortality rate was observed in the youngest age group (60–70 years: 10.9% vs. 5.7%; OR: 2.05, 95% CI: 1.30–3.24), decreased with age, and became non-significant in the oldest age group (≥90 years: 16.2% vs. 17.3%; OR: 0.93, 95% CI: 0.80–1.08). Significant differences were found for digestive system disorders (OR: 1.59; 95% CI: 1.15–1.89), cardiovascular and cerebrovascular disorders (OR: 1.51; 95% CI: 1.30–1.75), endocrine, nutritional, and metabolic diseases (OR: 1.42; 95% CI: 1.06–1.90), and pneumonia (OR: 1.20; 95% CI: 1.04–1.37), as well as for all clinic departments except for geriatric departments. Conclusion: The excess mortality rate was highest in younger age groups, where the general mortality and complication rate is relatively low in the general population. Appropriate approaches are needed, especially in non-geriatric wards.

## 1. Introduction

In times of demographic changes, the number of patients affected by Alzheimer’s disease and other related dementia disorders will tremendously increase [1]. This rising prevalence will also have an impact on hospitals, which will face growing admissions for PlwD and a higher demand for managing their complex in-hospital treatment and care processes [2].

A study by Sampson et al. [3] investigated the prevalence of dementia diseases in older people above the age of 70 undergoing emergency admissions, revealing that more than 42% of admitted patients had dementia. These results were in line with a cross-sectional study of patients aged 65 and older in randomly selected general hospitals in Germany [4], confirming that 40% of patients older than 65 years of age had mild cognitive impairment or dementia. These patients were more often treated for dehydration, electrolyte disturbances, urinary tract infections, contusions, and bone fractures, as well as for symptoms and findings of an unknown nature [5]. Over the last two decades, Blandi et al. [6] observed an increased mortality among PlwD admitted to hospital. However, the study also provided evidence of an increasing appropriateness of these hospitalizations.

Patients with severe impairments, such as dementia, need special care. A significant aspect that demands attention is the in-hospital mortality of this population group. An observational cohort study from 2010 linked data from three Dutch national registers that included 40,500 patients between 60 and 100 years demonstrated that besides being of the male sex and having a higher age, having Alzheimer’s disease increased the in-hospital mortality risk [7]. Later studies confirmed this higher likelihood of in-hospital mortality in patients with Alzheimer’s disease or other related dementia disorders with comparable results [8,9]. Therefore, dementia represents a predictor of in-hospital mortality for hospitalized individuals. Based on these findings, Kaur et al. [10] developed and validated a prognostic model to predict one-year all-cause mortality in dementia. This model can be used to identify patients with dementia who are at high-risk of one-year all-cause mortality to facilitate timely referrals to palliative care.

Understanding in-hospital mortality in dementia patients requires examining various contributing factors. Dementia often occurs in combination with other diseases, for example, respiratory failure, acute renal failure, hemorrhagic stroke, bloodstream infection [9], and delirium superimposed on dementia [11]. Also, Houttekier et al. [12] revealed that almost half of all those with dementia died from pneumonia, and a quarter of those living in long-term care settings died in a hospital.

Dementia leads to a compromised functional state and could raise the level at which intensive care measures are deemed necessary. Alternatively, the living will of such patients might explicitly rule out intensive care measures. Consequently, the percentage of dementia patients receiving intensive care measures might be lower, which could contribute to higher mortality rates.

However, there is missing evidence about the in-hospital mortality of PlwD across age groups, various primary admission diagnoses, and specific hospital wards. Knowledge about the impact of these factors on in-hospital mortality for PlwD would be of vital importance to initiate dementia-specific measures to prevent in-hospital deaths in this population group. The aim of this study was to evaluate the association between dementia and in-hospital mortality. The study tried to answer the question of whether in-hospital mortality rates of PlwD differ from those of patients without dementia across different age groups, clinical departments, and admission diagnoses.

## 2. Methods

### 2.1. Data Source

This multicenter case-control study was based on a hospital database (Company: IQVIA, Frankfurt, Germany), which contains data from fourteen hospitals across Germany, including specialized hospitals, primary care hospitals, maximum care, standard care, and university hospitals. The dataset describes a standardized data format that the hospitals transmit to the Reimbursement Institute for Hospitals (InEK, Siegburg, Gemany) following §21 of the Hospital Compensation Act (KHEntgG). The individual treatment episodes included in the §21 dataset of a case are grouped using special grouper software developed by 3M Health Information Systems and IQVIA. In addition, the export files generated by the software are anonymized (e.g., case and patient number) for data protection reasons before transmission. For this study, the complete data were available as Excel files for further analyses.

### 2.2. Study Population

The case-control study included all hospitalized patients aged ≥ 60 with admissions between January 2019 and July 2023. Only the last hospitalization was included in the analysis when patients were hospitalized more than once in the study period in order to avoid underestimating the mortality prevalence. Patients were grouped into patients with and without dementia diagnosis based on comorbidity (ICD-10: F00-F03, G30).

### 2.3. Study Outcome

**Figure 1:**
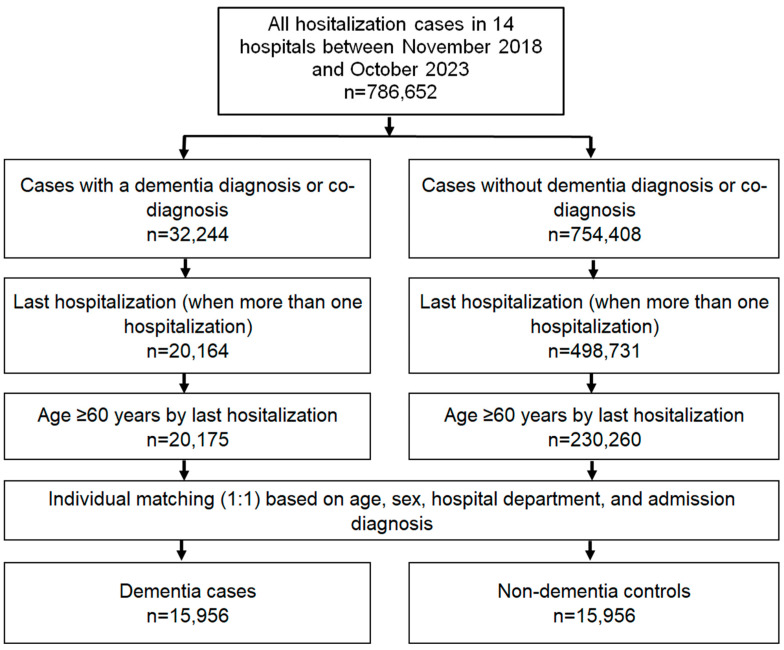
Selection of study patients.

The outcome of the study was the association between dementia and in-hospital mortality. Mortality was determined from medical records, as the dataset contains death as one of the discharge types. Patients with dementia were individually matched (ratio 1:1) to patients without dementia by age, sex, hospital department (internal medicine, geriatrics, surgery, cardiology, gastroenterology, and others), as well as admission diagnoses (see Figure 1, Supplementary Table S1). Patients were matched on all criteria simultaneously. The proportion of deceased patients was calculated for patients with and without dementia in total as well as stratified by age groups (60–69, 70–79, 80–84, 85–89, and 90+ years), women, men, and admission diagnoses.

### 2.4. Admission Diagnoses

Admission diagnoses were summarized in diagnosis categories based on ICD-10 codes, which included injuries and fractures (S00-S99), cardiovascular and cerebrovascular disorders (G45, I00-I99), pneumonia (J12-J18), digestive system disorders (K20-K95), endocrine, nutritional, and metabolic diseases (E00-E89), infectious and parasitic diseases (A00_B99), urinary tract infections (N30, N39.0), cancer (C00-C97), neurological diseases (G00-G99 excl. G45), musculoskeletal system disorders (M00-M99), acute renal failure (N17), and chronic obstructive pulmonary disease (COPD, J44). All other disorders were rarely documented as admission diagnoses and were instead summarized as “others”. The diagnosis groups were based on the frequency of these diagnoses in the database. For example, cancer diagnoses were relatively rare and were taken together (C00-C97); since pneumonia was very frequent, it was analyzed separately. 

### 2.5. Statistical Analyses

Differences in the sample characteristics and diagnosis prevalence between dementia cases and non-dementia controls were compared using the T-test for continuous variables, the McNemar test for categorical variables with two categories, and the Stuart–Maxwell test for categorical variables with more than two categories.

To assess the associations between dementia and in-hospital mortality, univariable logistic regression analyses were conducted. In-hospital mortality (yes, no) was the dependent variable, and dementia (yes, no) was the predictor of interest. Models were conducted for the total population and by age groups, sex, and admission diagnosis categories and wards. The results of the logistic regression models were given as the odds ratio (OR) for dementia compared to non-dementia patients. Due to multiple comparisons, *p*-values < 0.005 were considered statistically significant only. All analyses were performed using SAS 9.4 (SAS Institute, Cary, NC, USA).

## 3. Results

### 3.1. Baseline Characteristics

The present study included 15,956 patients with dementia (cases) and 15,956 without dementia (controls). Among dementia cases, the most frequent dementia diagnosis was unspecified dementia (70.6%), followed by vascular dementia (13.7%), and Alzheimer’s dementia (10.9%). The basic characteristics of study patients are displayed in Table 1.

The mean age was 83.9 years (standard deviation (SD): 6.5 years). A total of 60.7% of patients were female. A total of 27.0% were treated in internal medicine, 19.0% in surgery, 15.0% in geriatrics, 8.9% in cardiology, 8.7% in gastroenterology, and 22.4% in other departments. The most frequent admission diagnoses were injuries and fractures (21.9%), followed by cardiovascular and cerebrovascular disorders (17.6%), pneumonia (11.4%), digestive system disorders (8.8%), and endocrine, nutritional, and metabolic diseases (8.0%). The mean length of stay was significantly higher in patients with dementia compared to healthy controls (10.1 vs. 9.9, *p* = 0.002) (see Table 1).

### 3.2. Prevalence of In-Hospital Mortality

**Figure 2:**
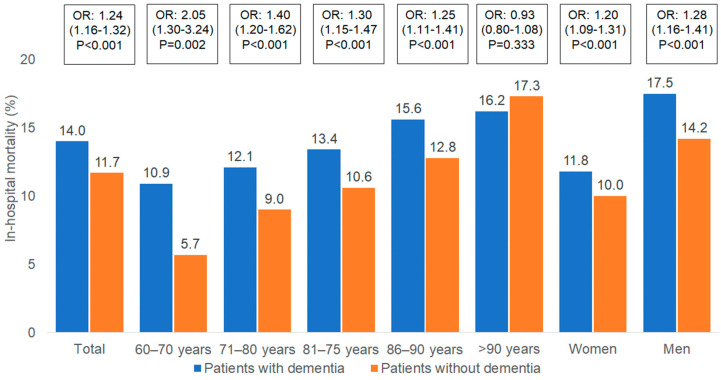
In-hospital mortality in patients with and without dementia by age and sex.

Figure 2 shows the proportion of deceased patients with and without dementia. Overall, in-hospital mortality was significantly higher in patients living with dementia compared to matched controls (14.0% vs. 11.7%, *p* < 0.001). The difference between in-hospital mortality rates for patients living with dementia and patients without dementia was highest in the youngest age group (60–70 years: 10.9% vs. 5.7%, *p* < 0.001), and constantly decreased in higher age groups, resulting in the lowest and non-significant difference in the population group at the age of 90 or older (16.2% vs. 17.3%). Also, in-hospital mortality rates significantly differed between groups of men and women. Mortality rates were higher in men (17.5% in PwD vs. 14.2% for controls) than in women (11.8% in PwD vs. 10.0% controls), and differences in in-hospital mortality rates between patients living with dementia were also higher in men than in women.

### 3.3. Association between Dementia and In-Hospital Mortality

Based on a multivariable regression model, dementia was significantly associated with higher in-hospital mortality (OR; 1.24; 95% CI: 1.16–1.32) irrespective of the primary diagnosis (Table 2). The odds for in-hospital mortality for patients living with dementia were much higher in the age group of 60–70 years (OR: 2.05; 95% CI: 1.30–3.24), and decreased with age reaching, the non-significant OR: 0.93 (95% CI: 0.80–1.08) in the age group of those older than 90 years.

Considering the admission diagnosis, an association between dementia and mortality was observed in patients admitted with digestive system disorders (OR: 1.59; 95% CI: 1.15–1.89), cardiovascular and cerebrovascular disorders (OR: 1.51; 95% CI: 1.30–1.75), endocrine, nutritional, and metabolic diseases (OR: 1.42; 95% CI: 1.06–1.90), and pneumonia (OR: 1.20; 95% CI: 1.04–1.37) as primary diagnosis of the in-hospital stay (see Table 2). For all other disease groups, no associations between dementia and in-hospital mortality were observed.

By differentiating the hospital wards, we found a significant association between dementia diagnosis and in-hospital mortality in patients treated in the gastroenterology (OR: 1.39; 95% CI: 1.11–1.73), internal medicine (OR: 1.31; 95% CI: 1.17–1.47), cardiology (OR: 1.30; 95% CI: 1.06–1.59), and surgery department (OR: 1.20; 95% CI: 1.00–1.43). There was no association between in-hospital mortality and dementia for patients treated in the geriatric department (Table 3).

## 4. Discussion

This case-control study evaluated the in-hospital mortality of 15,956 hospitalized patients with dementia and 15,956 hospitalized patients without dementia over age, clinical department, and main admission diagnosis groups. The in-hospital mortality difference between patients living with dementia and patients without dementia was highest in the youngest age group of 60 to 70 years of age (OR 2.05), constantly decreased with a higher age, and vanished in the age group of those 90 years and older. Also, a higher in-hospital mortality was detected for patients being mainly admitted with digestive system disorders, cardiovascular and cerebrovascular disorders, endocrine, nutritional, and metabolic diseases, and pneumonia as the primary diagnosis of the in-hospital stay. There was no association between in-hospital mortality and dementia for patients treated in the geriatric department. However, differences in the mortality were observed for patients from the gastroenterology, internal medicine, cardiology, and surgery departments.

Previous studies reported an in-hospital mortality risk between 11% and 19%, depending on the primary admission diseases [3,13,14]. Therefore, the overall in-hospital mortality rate of this study (14%) aligns with these studies. Older individuals generally exhibit higher susceptibility to diseases, complications, and functional impairments, significantly affecting their survival probability [15]. The presence of multiple chronic conditions, as is the case in patients with dementia diseases [16], causes a decline in physiological reserve, contributing to the complexity of handling in-hospital treatment and care in this population group. Taudorf et al. [17] showed that mortality in the patients living with dementia was more than 2-fold higher than in the general population, even after adjusting for comorbidities, suggesting that dementia, per se, contributes to increased mortality.

However, our study also demonstrated differences in the overall in-hospital mortality rate over different age groups, indicating that the excess mortality, defined as the difference in the mortality rate between patients living with dementia and matched controls without dementia, was highest in the youngest group (60 to 70 years). The correlation between age and hospital mortality is multifaceted, whether in or out of hospital, and mortality increases with age [18]. The study of Walicka et al. [19] evaluated in-hospital mortality in a large population, confirming that the mean odds ratio for in-hospital mortality increased with the age of patients, reaching a 229-fold higher rate in the ≥ 95 years age group as compared to the 18–24 age group. Comparing the observed age groups of Walicka et al. [19], there was an in-hospital mortality rate of 4.1% in patients aged 65 to 74 that increased to 7.5% in those aged 75 to 84, 15.0% aged 85 to 94, and 26.4% aged 95 and older. Our results confirmed this general trend of an increasing in-hospital mortality rate in higher age groups.

However, and most importantly, our results revealed that differences in the mortality rates between patients with dementia and patients without dementia were highest in the youngest observed age group of patients at the ages of 60 to 70. The mortality rate differences declined in higher age groups, becoming non-significantly different in patients at the age of 90 years or older. Therefore, the impact of dementia and the disease-associated risk factors and complications on mortality can change with age, probably having a more pronounced effect on mortality in younger ages where the general mortality rate is lower and the likelihood of infections and complications, as explained above, is higher as compared to the patients without dementia at the same age. In line with this, the possibility for in-hospital complications in the general population grows with increasing age [15]. Thus, our results revealed that the interplay of various health conditions and their relative contributions to in-hospital mortality can shift throughout aging, and the causes of death in individuals with dementia may vary at different age groups. This was confirmed by our department-specific analyses, revealing significant associations between dementia and mortality for all departments, except for those treated in the geriatric department, in which significantly older people were admitted and treated. 

However, it is important to mention that age alone is not the sole determinant of in-hospital mortality. Overall health status, comorbidities, the quality of medical care services, and social support also play pivotal roles. Further research is urgently needed to evaluate the impact of all these determinants on in-hospital mortality in this population group.

Another important finding in our study are the significant differences that were found for digestive system, cardiovascular and cerebrovascular disorders, endocrine, nutritional, and metabolic diseases, and pneumonia diagnoses. These diseases often require intensive care measures. Often, the living will of dementia patients might explicitly rule out intensive care measures. However, no data were available on patients’ living wills to check this hypothesis.

Also, a more holistic approach of clinical care is essential to effectively meet the diverse and complex needs of hospitalized patients living with dementia. There is various scientific literature about dementia-friendly initiatives that are most commonly implemented in geriatric wards [20,21,22]. Such approaches were implemented based on a more person-centered approach and various architectural criteria and interior design elements as key features to meet the challenges faced by patients with dementia during hospitalization [22]. However, such approaches do not mainly focus on reducing in-hospital complications and risk factors that could cause the higher in-hospital mortality in patients living with dementia, and are often implemented in geriatric wards, where we have seen similar in-hospital mortality rates between PlwD and patients without dementia. Therefore, the impact of such holistic and dementia-focused approaches should be evaluated, especially in younger population groups that were treated in non-geriatric wards, like internal medicine departments.

Our findings are of interest for a better understanding of the differences in the in-hospital mortality of patients living with dementia compared to patients without dementia across age, department, and admission disease groups. The study results indicated that the excess in-hospital mortality due to dementia can significantly differ, being highest in the youngest patient group, where the mortality rate of the elderly population or patients without dementia was low and dementia-related in-hospital complications, such as complications, could have the highest impact. This mortality rate difference declined with age and vanished in patients at the age of 90 or older, where the mortality rate and possibility for complications in the general, population of patients without dementia is high, reducing the impact of dementia on in-hospital mortality. Especially non-geriatric wards treating, on average, younger patient groups compared to geriatric wards, are commonly affected by a higher excess in-hospital mortality rate of patients living with dementia. Therefore, appropriate approaches to prevent hospital admission, better access to clinical care, and tailored management of comorbidities, complications, and acute events to prevent poor clinical outcomes in this population are vital. Further research should evaluate the impact of such innovative approaches on adverse in-hospital outcomes and mortality, especially in relatively younger patient groups where the highest reduction potential can be achieved.

## 5. Limitations

The present study also displays several limitations that should be acknowledged. Firstly, the diagnoses and co-diagnoses relied exclusively on the ICD-10 classification. Secondly, data from 2019 to 2023 were included, a period where the COVID-19 pandemic had a tremendous effects on hospital care and patient deaths, especially in the elderly population. Therefore, the demonstrated results may be limited to these effects and not generalizable to a situation without COVID-19 interference. Thirdly, no data were available on lifestyle variables, such as residence in nursing homes or family situations. Fourth, no data were available on medication to analyze the impact of several drugs on mortality. Additionally, as the dataset only contains information from hospitals, the study results cannot be extrapolated to the outpatient population. Also, socio-demographic, socio-economic, or clinical variables of the patients would have been useful to improve our models. However, due to the fact that this analysis was based on claim data, none of these variables exist, which may have affected the demonstrated results, leaving important associations uncovered. Furthermore, as only the last hospitalization was used for analyses, the mortality rate in this study may be slightly overestimated. Finally, patient treatment and care and, thus, death, also depends on the quality of care of a specific hospital. Including the hospital ID and region as clusters would help to adjust for such differences. However, due to data protection regulations, no information about the region or the hospital ID was available, limiting the generalizability of the demonstrated results. Furthermore, the data analyses were based on a 1:1 matching procedure of PlwD with patients without dementia, which excluded some individuals living with dementia. However, a matching of all individuals would result in differences between the samples. However, the reduced sample used for this analyses limits the generalizability of the presented results. Finally, the study’s reliance on administrative data might limit the ability of this study to capture the complexity of dementia and its impact on mortality.

## Figures and Tables

**Table 1:** Baseline characteristics of the study sample after 1:1 matching.

	Patients with Dementia (n = 15,956)	Patients without Dementia (n = 15,956)	*p*-Value
Mean age			
Mean age (SD)	83.9 (6.5)	83.9 (6.5)	1.000
60–70 years, n (%)	531 (3.3)	531 (3.3)	
71–80 years, n (%)	3732 (23.4)	3732 (23.4)	
81–75 years, n (%)	5010 (31.4)	5010 (31.4)	1.000
86–90 years, n (%)	4297 (26.9)	4297 (26.9)	
>90 years, n (%)	2386 (15.0)	2386 (15.0)	
Gender, n (%)			
Female	9691 (60.7)	9691 (60.7)	
Male	6265 (39.3)	6265 (39.3)	1.000
Hospital department, n (%)			
Internal medicine	4258 (27.0)	4258 (27.0)	
Surgery	2991 (19.0)	2991 (19.0)	
Geriatrics	2368 (15.0)	2368 (15.0)	1.000
Cardiology	1398 (8.9)	1398 (8.9)	
Gastroenterology	1370 (8.7)	1370 (8.7)	
Other departments	3571 (22.4)	3571 (22.4)	
Length of hospital stay			
Mean length of stay (SD)	10.1 (9.4)	9.9 (9.6)	0.002
Main diagnosis, n (%)			
Injuries and fractures	3487 (21.9)	3487 (21.9)	1.000
Cardiovascular and cerebrovascular disorders	2810 (17.6)	2810 (17.6)	
Pneumonia	1818 (11.4)	1818 (11.4)	
Digestive system disorders	1409 (8.8)	1409 (8.8)	
Endocrine, nutritional, and metabolic diseases	1280 (8.0)	1280 (8.0)	
Infectious and parasitic diseases	858 (5.4)	858 (5.4)	
Urinary tract infections	767 (4.8)	767 (4.8)	
Cancer	446 (2.8)	446 (2.8)	
Neurological diseases	321 (2.0)	321 (2.0)	
Musculoskeletal system disorders	260 (1.6)	260 (1.6)	
Renal failure	280 (1.8)	280 (1.8)	
COPD	177 (1.1)	177 (1.1)	
Other disorders	2034 (12.8)	2034 (12.8)	

**Table 2:** Association of dementia with the in-hospital mortality in patients hospitalized for different diseases (univariable logistic regression).

Main Diagnosis	In-Hospital Mortality among Patients with Dementia (%)	In-Hospital Mortality among Patients without Dementia (%)	OR (95% CI)	*p* Value
Total	14.0	11.7	1.24 (1.16–1.32)	<0.001
Injuries and fractures	6.5	7.2	0.89 (0.74–1.08)	0.236
Cardiovascular and cerebrovascular disorders	17.4	12.2	1.51 (1.30–1.75)	<0.001
Pneumonia	35.0	31.0	1.20 (1.04–1.37)	0.011
Digestive system disorders	12.0	8.5	1.58 (1.15–1.89)	0.002
Endocrine, nutritional, and metabolic diseases	9.1	6.6	1.42 (1.06–1.90)	0.019
Infectious and parasitic diseases	21.1	18.4	1.18 (0.93–1.50)	0.163
Urinary tract infections	8.3	6.3	1.36 (0.93–2.01)	0.118
Cancer	17.5	13.5	1.36 (0.95–1.97)	0.096
Neurological diseases	7.8	7.8	1.00 (0.56–1.78)	1.000
Musculoskeletal system disorders	3.9	2.3	1.69 (0.61–4.73)	0.315
Renal failure	28.2	24.6	1.20 (0.83–1.75)	0.338
COPD	21.5	14.7	1.59 (0.92–2.75)	0.099
Other disorders	6.1	5.1	1.19 (0.91–1.56)	0.197

**Table 3:** Association of dementia with the in-hospital mortality in patients hospitalized for different hospital wards (univariable logistic regression).

Main Diagnosis	In-Hospital Mortality among Patients with Dementia (%)	In-Hospital Mortality among Patients without Dementia (%)	OR (95% CI)	*p* Value
Internal medicine	19.4	15.5	1.31 (1.17–1.47)	<0.001
Surgery	9.7	8.2	1.20 (1.00–1.43)	0.046
Geriatrics	5.6	6.1	0.92 (0.72–1.17)	0.456
Cardiology	17.5	14.1	1.30 (1.06–1.59)	0.013
Gastroenterology	15.4	11.6	1.39 (1.11–1.73)	0.004
Other departments	14.9	12.8	1.20 (1.05–1.37)	0.009

## Data Availability

The datasets used and analyzed during the current study are available from the corresponding author on reasonable request. The data are not publicly available due to privacy reason.

## Supplementary Materials

The following supporting information can be downloaded at: https://www.mdpi.com/article/10.3390/brainsci14050455/s1, Table S1: Overview about diagnoses per group.
