# Supplementary material for: In-Hospital Mortality in Patients with and without Dementia across Age Groups, Clinical Departments, and Primary Admission Diagnoses
Source: Brain Sci. 2024 Apr 30;14(5):455. doi: 10.3390/brainsci14050455 (PMC11118056; doi:10.3390/brainsci14050455)
Supplement: Supplementary file 1 [file brainsci-14-00455-s001.zip › brainsci-2952823-supplementary.pdf]

Supplementary Table S1: Overview about diagnoses per group

**Main diagnosis**	**Number and proportion of patients with dementia (%)**	**Number and proportion of patients without dementia (%)**
**Cardiovascular and cerebrovascular disorders**	17.6	17.6
Heart failure	6.3	6.3
Ischemic stroke	3.0	3.0
Atherosclerosis	1.7	1.7
Primary hypertension	0.9	0.9
Myocardial infarction	0.9	0.9
Atrial fibrillation and flutter	0.9	0.9
Pulmonary embolism	0.5	0.5
**Digestive system disorders**	8.8	8.8
Gastrointestinal hemorrhage, unspecified	1.5	1.5
Paralytic ileus and intestinal obstruction without hernia	1.3	1.3
Cholelithiasis	0.9	0.9
Other functional intestinal disorders	0.8	0.8
Gastritis	0.5	0.5
Diverticular disease of intestine	0.5	0.5
**Endocrine, nutritional, and metabolic diseases**	8.0	8.0
Volume depletion	6.0	6.0
Diabetes mellitus Type 2	1.4	1.4
Other disorders of fluid, electrolyte and acid-base balance	0.6	0.6
